# Harm Reduction Text Messages Delivered During Alcohol Drinking: Feasibility Study Protocol

**DOI:** 10.2196/resprot.1970

**Published:** 2012-05-23

**Authors:** Karen Adell Renner, Natalie Walker, Varsha Parag, Ross McCormick

**Affiliations:** 1 General Practice and Primary Health CareSchool of Population HealthUniversity of AucklandAucklandNew Zealand; 2 Clinical Trials Research UnitSchool of Population HealthUniversity of AucklandAucklandNew Zealand

**Keywords:** harm reduction, alcohol, eHealth, mobile phone, brief intervention, smartphone

## Abstract

Background: Recent research using mobile phone interventions to address public health issues such as smoking, obesity, depression, and diabetes provides a basis for trialing a similar approach toward reducing the negative consequences of risky drinking. Objective: This feasibility study aims to recruit drinkers between 18–34 years to a website where they will design and enter their own personal messages (repeating or one-off) to be sent to their mobile phones when they are drinking to remind them of their pre-drinking safety intentions. Methods/Design: Participants in the treatment group will have access to the messaging function for 3 months and will be compared to a control group who will have 3 months access to a web chat site only. Data collection will occur at baseline, 3 months, and 6 months. The primary outcome is a change in unintended negative consequences from drinking at 3 months. Secondary outcomes include the acceptability of the intervention to this population, recruitment rate, participant retention, reduction in alcohol consumption, and the self-motivation discourse in participant messages. Discussion: Existing alcohol interventions in New Zealand attempt to reduce alcohol consumption in the population, but with little effect. This study aims to target unintended negative consequences resulting from drinking by empowering the drinkers themselves to deliver safety messages during the drinking session. If proven effective, this strategy could provide a cost-effective means of reducing the public health burden associated with risky drinking. Trial Registration: Australia and New Zealand Clinical Trials Register (ANZCTR): ACTRN12611000242921

## Introduction

New Zealand statistics about the unintended negative consequences of drinking show that young drinkers do not heed public health messages sufficiently [1]. There are numerous immediate problems caused by risky drinking, including acts of violence, driving under the influence, unintended sex, hangover, cognitive impairment, physical and verbal assault, physical accidents, relationship problems, loss of productivity, compromised mental health, and increased burden on medical and legal services [2,3]. For example, a study of the New Zealand drinking population in 2009 found that the risk of alcohol-associated accident and injury was almost 50% for those younger than 24 years compared to a 20% risk among drinkers of all ages [4].

Risky drinking can be defined generally as drinking to drunkenness [5], or quantified as more than 6 standard drinks for men (4 for women) in one drinking session [2]. In the 2007–2008 New Zealand Health Survey, 25% of respondents were identified as weekly risky drinkers. Most weekly risky drinkers were between 18–24 years (33.8% of males and 18.8% of females) followed by those between 25–34 years (16.3% of males and 12.6% of females). Young women in the 16–17 year age group were more likely to be weekly risky drinkers (16.4%) compared to young men in this same age group (9.0%) [2]. In New Zealand, the two demographic groups that engage in weekly risky drinking most frequently are between 18–24 years and 25–34 years.

Brief interventions, such as an assessment of drinking behavior and discussion about the consequences and how to change, have traditionally been left to health professionals in the public sector [6-8] or in the university campus environment [9-12]. Web-based alcohol interventions are now gaining popularity by providing information and strategies on reducing consumption and keeping safe [13-15]. Although medical and web interventions have shown some measure of success [11,16-20], particularly in the university population[12,21,22], the continuing alcohol problem among those between 18–34 years is an indication that alternate approaches are needed.

Computer- and mobile phone-based technologies have been utilized effectively in a number of health interventions, such as smoking, diabetes, obesity, and depression prevention [23-27]. The rationale behind mobile phone interventions for smoking is to send messages or relevant video to smokers’ phones when they are most likely to be tempted to smoke as a reminder of cessation intentions [24]. Similarly, a text message received while drinking could serve as a reminder of safety intentions and counter the distraction of drinking “buddies” and the drinking environment [28]. Engaging the participants in planning their own messages could add to the intervention effect due to the positive correlation between health behavior change and personally defined goals and plans [29-31]. The receipt of a planned, self-designed message sent at appropriate pre-scheduled drinking times could potentially resonate with a message recipient (whose judgment is clouded by alcohol) and result in safer behavior [32]. An additional rationale behind the drinker designing their own message (versus messages designed by a third party) is one of ontology. Researchers traditionally use focus group discussions with representatives of the target population to construct the messages used in health interventions. However, the brain of the drinker, dulled by the sedative effect of alcohol, requires a message that fully resonates within their memory [28]. It is our view that such a message can only be written by the drinkers themselves.

We have designed a feasibility study to look at whether drinkers between 18–34 years are able to fulfill their intentions of drinking safely and/or consuming less alcohol if they receive self-generated reminders of those intentions via mobile phone text messages when drinking.

The study aims to address several gaps in the alcohol brief-intervention literature, namely (1) delivery of a brief intervention by non-health professionals; (2) use of text messages as a prompt to moderate drinking and implement intentions to keep safe while drinking; (3) use of an intervention during alcohol consumption; (4) use of an intervention designed and controlled by the participants themselves; (5) how text message-prompted smoking cessation translates to the field of alcohol consumption; and (6) an insight into the self-motivation talk of drinkers.

The planned intervention puts the power and opportunity for change into the hands of those who are most at risk from the consequences of risky drinking. The intervention delivery mechanism, the mobile phone, is one that the younger drinking population is extremely comfortable using and it has been shown that they use it to transmit health information [33]. In New Zealand, 90%–94% of young people between 18–34 years own a mobile phone [34]. Changing health behaviors requires motivation, volition, and a sense of personal efficacy [30,35-37]. The process of planning the messages to be sent to their mobile phones may increase the participants’ sense of self-efficacy. Their likelihood of succeeding in their intentions could also be increased by their proactive planning of the message and scheduling of the delivery [35]. The delivery of the message during the act of drinking should cut across the physical effects of alcohol and the social environment, thereby, increasing the probability of the participant taking action on their intentions [28,38,39]. In addition, participants can respond to their own changing needs by changing the messages and message delivery times at any time. This responsiveness has been extended by a smartphone application, available for the iPhone and the Windows Phone 7, which will enable messages to be created or edited without direct access to the website. The act of planning the text delivery times and messages aims to empower the participant and add to the salience and recognition of the messages when received on their phone.

Before a full, randomized controlled trial of this intervention can be undertaken, a feasibility study needs to be run. To this end, the website and smartphone apps have been developed and tested to ensure that the website information, question format, and data collection align with the Checklist for Reporting Results of Internet E-Surveys (CHERRIES) protocol [40].

## Methods

### Objectives

The long-term objective of the feasibility study is to trial concepts needed to inform a randomized controlled trial (RCT) which will assess whether a mobile phone harm reduction strategy can reduce the number of unintended consequences resulting from a drinking session.

The primary objectives of this study are the acceptability of the intervention, a change in amount of alcohol consumed and/or a change in alcohol-related consequences, the self-talk of this population in relation to alcohol and safe drinking, and the recruitment rates and loss to follow-up over time. Such data will inform sample size calculations and the design of a subsequent RCT.

### Study Design

This is a parallel group randomized controlled trial.

### Study Population

The study population will be recruited within the Auckland Council region using media advertising, including radio and newspapers. Corporate businesses, bars, and clubs will be contacted for permission to display recruitment posters on their premises. Tertiary campuses in the study region will also be targeted through student radio, flyers, and posters, and student email lists from the campuses (where they can be sourced). Participants will be invited to sign up on the study website where they can access further information about the study. They will be asked 4 key questions to determine whether they fulfill the inclusion criteria. Those not eligible for the study will be thanked for their time, and they can sign up to receive a copy of the study results and/or notification of any subsequent trials.

Participants will not be paid for their participation, but will be entered into a monthly draw for mobile phone top-up vouchers.

### Inclusion Criteria

Participants will be between 18–34 years, have experienced at least one unintended consequence from drinking in the previous 3 months as measured by the selected Screening Test: Young Adult Alcohol Problem Severity Test (YAAPST), have access to the Internet, and own a mobile phone on any plan. Non-drinkers will not be excluded from participation as they may experience unintended consequences due to their association with drinkers. Non-drinkers may socialize with drinking friends and be the “responsible” person in the group. As such, they may want to use the text message service to remind themselves of this responsibility. We did not want to exclude non-drinkers from the study as non-drinkers can be caught up in unintended negative consequences when out with drinking friends. The Alcohol Use Disorders Identification Test alcohol consumption questions (AUDIT-C) scores will identify these non-drinkers and we will control for them in the statistical analysis.

There will be no limit to the number of participants. Recruitment will remain open for 8–10 months.

### Exclusion Criteria

Adults will be excluded if they have not experienced unintended consequences from drinking, they are outside the target age range, or they do not have their own mobile phone or access to the Internet.

### Blinding and Randomization Allocation

Participants will be allocated 2:1 to the control and intervention groups, respectively, to ensure adequate numbers for study comparison. There is evidence of a high level of attrition in the control group in Internet-based interventions. Therefore, we chose the 2:1 allocation to reduce the risk of control group dropout. The researchers will be blinded to treatment allocation. Participants fulfilling the inclusion criteria will be asked to consent to the study and answer demographic, drinking, and consequences questions before gaining on-going access to the website. Participants will be randomized to each of the two groups by computerized central randomization using stratified blocks (block size = 6). Two stratification factors will be used: gender (male/female) and age (≤ 24 years and > 24 years) to ensure balance in these key prognostic factors between the intervention and control groups. Once randomized, intervention participants will receive an appropriate email with instructions and reminders for setting up and accessing their messages.

### Study Intervention

#### Intervention Group

The study website will enable participants to create the messages they wish to have sent to their mobile phones, at the times they schedule. These messages might relate to their strategies to reduce alcohol consumption or to keep safe during and after drinking. By default, the messages will be repeated weekly based on the initial day of the week and time specified by the participant. Participants will be able to create once-only messages by entering a specific date. All messages will continue to be sent, as per the participant schedule, until they are deactivated by the participant. Participants can change their messages and schedules at any time. They will also have access to the website community pages (outlined below).

**Figure 1:**
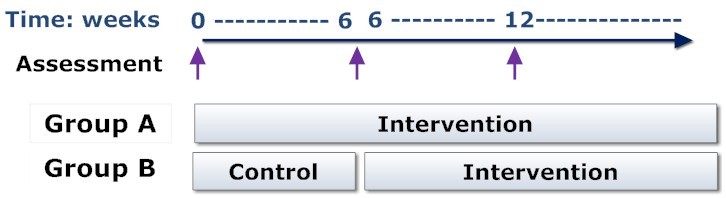
Study intervention and control groups.

Participants who own an iPhone or Windows Phone 7 will also be able to download an application to their phone to enable them to control the messages from their mobile phone. The intervention group will have access to the intervention for six months. Figure 1 illustrates the study design.

#### Control group

Participants in this group will have access to a simple website for 3 months, where they can post messages about any topic of interest to them and participate in community discussions. The community pages will also have links to useful websites providing information on drinking safely and participants will receive a text message as these links are updated. This group will understand that they are in the control group and, after 3 months, they will receive an email or text message inviting them to access the intervention.

There will not be a limit placed on the number of messages created by the participants or on the frequency with which these messages can be sent to their mobile phones. A log of messages sent and a log of message content/schedule changes will be recorded from the website and will inform statistics on intervention usage.

### Baseline Assessments

The following data will be collected via the study website from all participants at baseline and saved directly into the Microsoft SQL Server database 2008 Enterprise R2.

#### Demographic Information

Information about gender, ethnicity, employment status, age of first drink and first drunkenness, and readiness to change drinking habits [41] will be collected.

#### Drinking History

Items 1–3 of the AUDIT-C will be used to assess alcohol intake [42]. This measure has been well validated in university students between 18–21 years [43-46], both male [47,48] and female [49]. Due to its brevity, the AUDIT-C has good utility as a computerized version [50] and when incorporated into general health risk questionnaires [51]. One limitation of the AUDIT-C is that Item 2, which provides the scale for number of alcoholic drinks consumed on a typical day, uses consumption ranges. To identify small changes in drinking habits [48] a question on typical daily consumption of each of wine/champagne, beer, spirits, alcopops/ready-to-drink beverages (RTD), and “other” alcoholic beverages will be included [52-55].

#### Other Dependencies

Question 2 of the Alcohol, Smoking and Substance Involvement Screening Test (ASSIST) will be asked to gauge other dependencies within this population. Previous research has shown a relationship between dependencies and difficulty in reducing drinking/negative consequences [56-58]. The ASSIST correlates well with the AUDIT (*r *= .82) when assessing alcohol as the substance of abuse [56].

#### Consequences Measure

The YAAPST was developed for use with American university students between 18–21 years [59], and is a sensitive measure for mild alcohol-related consequences, such as hangover, feeling sick, being late for work/school, etc [60]. The YAAPST also includes items on unintended sex and driving while drunk [61-68]. The measure can be scored as a lifetime occurrence of consequences, past year frequency, or past year severity [69]. There is good reliability for recall over a 12-month period [70]. Therefore, we expect similar reliability over a shorter period of time. This study will compare consequences at baseline, 3 months, and 6 months. To make the 3- and 6-month comparisons and avoid crossover of data, we will limit each recall period to 3 months.

One of the aspects that we will look at is whether the assessment items can be reduced and still collect the data we need. The intervention sign-on process tracks the point at which potential participants drop out before sign-up and we will be reviewing this for dropout particularly at the assessment points.

### Primary Outcomes

Primary outcomes will be collected at 3 and 6 months and include:

1. Whether the intervention engaged the interest of the participants sufficiently to make them proactive users of the mobile phone messages.

2. The acceptability of the intervention as measured by recruitment and dropout rates.

3. Safety, assessed from participant feedback and by measuring reported website misuse such as spamming others. Participant time from sign-up to last message sent until 6 months will be measured to provide an indication of the longevity of the program.

In addition, a change in unwanted consequences reported by participants in the intervention group between baseline and 6 months will provide an indication of the longevity of the intervention.

The quantitative outcomes of interest include a change in unwanted consequences and drinking. The initial question battery of AUDIT-C, YAAPST, and ASSIST will be repeated at 3 and 6 months. In addition, an open question asking the participant if they thought they had changed their drinking behavior in the previous 3 months, and if so, what changes they had made will be asked also.

The messages created by the participants will be analyzed to gain an understanding of the self-talk of this drinking population.

### Sample Size

This is a feasibility study and the results will be used to inform the required sample size for a well-powered randomized controlled trial. Every effort will be made to recruit as many participants as possible during the 6-week recruitment period.

### Withdrawal Criteria

There are no withdrawal criteria for this study. Participants wishing to drop out of the study can do so by returning to the website to turn off their messages. The termination of messages will be the proxy measure of dropout rate.

### Data Management

The lead researcher will design, validate, and verify the security of the databases used in this study. The SQL 2008 R2 databases will be managed by Starsoft Ltd, the writers of the software. All questionnaire data will be assigned range checks and will return instructions to the user as data items are entered. All data will be entered directly by the participants, who will enter their personal log-in and password to access their messages on the website [40].

### Data Analysis

This trial has been designed in collaboration with a senior statistician at the National Institute of Health Innovation, University of Auckland, who will continue to advise and assist with analyses of the trial data. The data from the Microsoft SQL database will be exported to an Excel spreadsheet and then imported into a statistical analysis package for integrity checks prior to analysis.

The qualitative data obtained from the messages created by the participants will be reviewed by, and discussed with, a senior academic qualitative researcher at the University of Auckland who has contributed to the theoretical underpinnings of the research design. The theory underlying this design is post-positivist, seeking to infer from a non-falsified hypothesis what is probably true or what will probably be useful. Analysis of qualitative data, namely the participant messages and feedback, will use a general inductive approach to gain an understanding of self-talk around alcohol and safety. As this is a feasibility study, participants will be asked to provide feedback on the usefulness of the intervention and on suggestions for improvement. The messages and comments will be assessed by thematic analysis using NVivo 9 from QSR International.

### Treatment Effects

The relationship between frequency of text message use and behavior change (ie, change in reported drinking and/or change in safety behaviors) will be investigated. For each group, paired *t *tests will be used to assess the treatment effect between baseline and 3 months. Change from baseline to 3 months in drinking and consequences scores will be analyzed using linear regression and the model will include group and the baseline values. To assess the effect of factors such as age, gender, and ethnicity, adjusted linear regression analysis will be conducted. And if there are sufficient participants, subgroup analysis will be conducted.

Data collected at 6 months will be analyzed according to the preceding 3-month protocol. If there are sufficient data, repeated measures analysis will be run to compare the two groups across the full 6-month follow-up. Scatter plots will be generated to show the change in scores over time. Change in consequences score will be plotted at each follow-up assessment against message intensity (volume of messages sent) and correlation between these two variables will be calculated. Alcohol/consequences data will be plotted against frequency/intensity of messaging to give an indication of the messaging relationship on outcomes.

### Procedure to Account for Missing Data

Analysis will be on an intention-to-treat (ITT) basis. Those lost to follow-up will be included in the data analysis and are defined as those not using the messaging function or those who fail to complete the outcomes measures at 3 months and 6 months. Missing data will be replaced by the last observation carried forward (LOCF) with no change assumed since the last message sent. If feasible, sensitivity analyses will be performed excluding participants with missing outcome data to assess the robustness of the study findings.

### Ethics

Ethics permission for this study has been received from the Central Regional Ethics Committee (ethics number CEN/11/03/010). As part of this ethical obligation, the researcher has committed to sharing research results and other issues of interest to Māori (indigenous New Zealanders) arising from this research in appropriate seminars. Maintenance of confidentiality and compliance with New Zealand’s privacy legislation will be obtained at the point of participant sign-up on the study website. Participation in the study is voluntary and participants will be free to withdraw at any time. Participants will be anonymous, signing into the website with a code name. Their password will be encrypted and held in the database in an encrypted format. Data will be entered, stored, and backed-up in a secure commercial database: Microsoft SQL 2008 R2 fully encrypts all data. A secure socket layer (SSL) certificate will protect data as it is entered into the website. Participants will be acknowledged in all study-related publications and presentations.

## Discussion

Alternate methods of prompting young drinkers to keep safe while drinking must be explored. These methods should resonate with the population they target. The relevance of the message and of message delivery is an important aspect of intervention success. The high ownership and uptake of mobile technology by young people in many countries [34] makes this medium an ideal vehicle for an intervention that aims to provide safety reminders at relevant times during drinking.

Success of this feasibility study will lead to a full RCT, preparatory to a publicly available website. In addition, an understanding of the self-talk of the study population may provide insight into other avenues that may be pursued to encourage safety while drinking. If the main RCT is successful, the intervention would provide a low-cost method of providing continuous support to risky drinkers.

## Abbreviations

ASSIST: Alcohol, Smoking and Substance Involvement Screening Test
AUDIT-C: Alcohol Use Disorders Identification Test alcohol consumption questions
CHERRIES: Checklist for Reporting Results of Internet E-Surveys
ITT: intention-to-treat
LOCF: last observation carried forward
RCT: randomized controlled trial
RTD: ready-to-drink
SSL: secure socket layer
YAAPST: Young Adult Alcohol Problem Severity Test
